# Unveiling a rare *BRAF* mutation in minimally invasive follicular thyroid carcinoma: A case report

**DOI:** 10.1097/MD.0000000000039364

**Published:** 2024-08-23

**Authors:** Po-Sheng Lee, Jui-Yu Chen, Li-Hsin Pan, Chii-Min Hwu, Jen-Fan Hang, Chin-Sung Kuo

**Affiliations:** a Section of Endocrinology and Metabolism, Department of Medicine, Taipei Veterans General Hospital, Taipei, Taiwan; b School of Medicine, National Yang Ming Chiao Tung University, Taipei, Taiwan; c Section of General Surgery, Department of Surgery, Taipei Veterans General Hospital, Taipei, Taiwan; d Section of Endocrinology and Metabolism, Department of Medicine, Taipei City Hospital Zhongxing Branch, Taipei, Taiwan; e Department of Pathology and Laboratory Medicine, Taipei Veterans General Hospital, Taipei, Taiwan; f Institute of Clinical Medicine, National Yang Ming Chiao Tung University, Taipei, Taiwan.

**Keywords:** *BRAF* V600E mutation, fine needle aspiration cytology, genetic molecular testing, thyroid cancer, thyroid nodule

## Abstract

**Rationale:**

Molecular testing is becoming more widely used; however, the accuracy of diagnostic testing remains a primary consideration, especially for molecular testing that detects specific mutations associated with cancers.

**Patient concerns:**

A 45-year-old female without documented comorbidities presented a thyroid nodule during a routine health examination. Initial evaluation revealed a 3.8-cm nodule in the left lobe of thyroid, classified as Bethesda System category III on fine needle aspiration cytology. Genetic molecular testing detected the *BRAF* V600E mutation via quantitative polymerase chain reaction assay, raising concern for papillary thyroid cancer (PTC).

**Diagnoses:**

The preoperative impression was PTC based on the detection of *BRAF* V600E mutation.

**Interventions:**

The patient underwent thyroidectomy as well as lymph node dissection with the expectation to treat PTC.

**Outcomes:**

The final pathology unexpectedly revealed minimally invasive follicular carcinoma. Confirmatory Sanger sequencing unveiled a novel sequence variation involving nucleotide duplication within the range of 1794 to 1802, a non-V600E *BRAF* mutation not previously reported in follicular thyroid carcinoma.

**Lessons:**

This case study demonstrates the clinical relevance of exercising caution in molecular testing and its interpretation of results. For genetic testing used for diagnostic purposes, rigorous validation or cross-checking using different methods should always be considered to ensure appropriate interpretation of molecular results.

## 1. Introduction

Thyroid cancer stands as the most prevalent among endocrine malignancies, with an age-standardized incidence rate of 15.46 per 100,000 person-years in Taiwan for the year 2019.^[1,2]^ Thyroid cancer commonly presents oncogenic genetic alterations, each associated with specific tumor types.^[3]^ Among these, the *BRAF* V600E mutation is the most frequently observed, primarily in papillary thyroid carcinoma (PTC).^[4]^ Before to the introduction of molecular testing, indeterminate thyroid nodules frequently necessitated diagnostic surgery.^[5,6]^ Molecular genetic testing performed before thyroid nodule surgery has demonstrated effectiveness in improving diagnostic accuracy, thereby contributing to the reduction of unnecessary thyroidectomies.^[6,7]^ However, the accuracy of diagnostic testing remains a primary consideration, especially for molecular testing that detects specific mutations associated with cancers. Herein, we introduce a case involving the Bethesda system category III thyroid nodule harboring a positive *BRAF* V600E mutation detected via quantitative polymerase chain reaction (qPCR) assay, followed by thyroidectomy and lymph node dissection.^[8]^ Nevertheless, the definitive pathology report yielded an unexpected diagnosis of indolent follicular thyroid carcinoma (FTC) accompanied by a rare non-V600E *BRAF* mutation, confirmed by Sanger sequencing. Moreover, we made a brief review of rare *BRAF* mutations in thyroid carcinomas.

## 2. Case presentation

The Institutional Review Board of Taipei Veterans General Hospital approved the study (IRB No: 2023-01-020CC), and the research was performed with written informed consent and in accordance with the Declaration of Helsinki.

A 45-year-old Taiwanese female came to the outpatient clinic because she had a left thyroid nodule for 2 years. She had no major underlying medical conditions. Taking her family history, her mother and younger sister were diagnosed with hypothyroidism.

**Figure 1.**
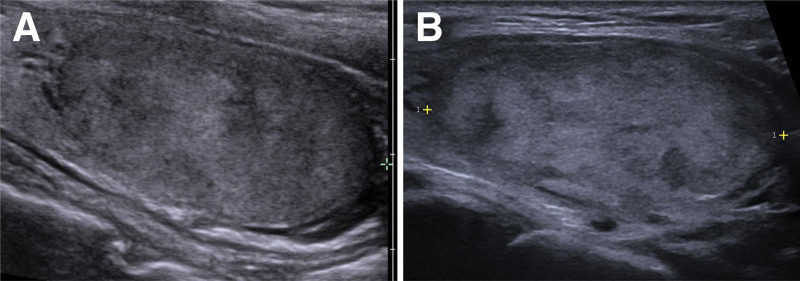
The ultrasonographic examination showed a 3.8-cm left thyroid mass, featuring solid composition, hypoechogenicity, wider-than-taller shape, smooth margin, and absence of echogenic foci categorized as thyroid imaging reporting and data system (TI-RADS) 4 (A) in Aug 2021 (B) in Jun 2023, with no discernible interval change.

The blood panel affirmed her euthyroid status without thyroid autoantibodies in terms of antithyroglobulin antibodies, antithyroid peroxidase antibody, and thyroid-stimulating immunoglobulin but elevated thyroglobulin level (Table 1). Sonographic examination of the thyroid revealed a 3.8-cm mass within the left thyroid lobe, featuring solid composition, hypoechogenicity, wider-than-taller shape, smooth margin, and absence of echogenic foci categorized as Thyroid Imaging Reporting and Data System^[9]^ 4 with no significant interval change in the recent 2 years (Fig. 1). Fine needle aspiration (FNA) was performed, with cytological analysis with enlarged nucleus and some microfollicular arrangement yielding a verdict of TBS category III: atypia of undetermined significance.^[8]^

**Table 1:** Thyroid function, tumor markers, and autoantibody data.

		Normal range
TSH	0.45 uIU/mL	0.27–4.20
FT3	2.6 pg/mL	2.0–4.4
FT4	1.15 ng/dL	0.93–1.7
TSI	<0.1 IU/O	<0.55
aTG	14.4 IU/mL	<115
aTPO	<9 IU/mL	<34
Thyroglobulin	700 ng/mL	<55
Calcitonin	<2 pg/mL	0–11.5
CEA	1.2 ng/mL	<5

aTG = antithyroglobulin antibodies, aTPO = antithyroid peroxidase antibody, CEA = carcinoembryonic antigen, FT3 = free triiodothyronine, FT4 = free thyroxine, TSH = thyroid-stimulating hormone, TSI = thyroid-stimulating immunoglobulin.

**Figure 2.**
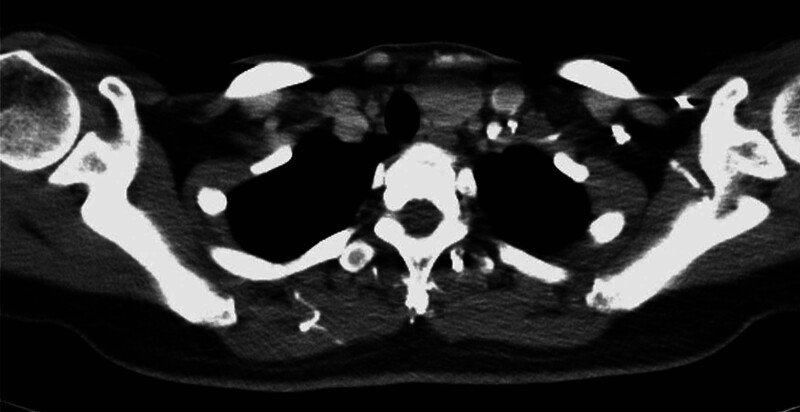
Computed tomography (CT) scan revealed a 3.8-cm mass situated within the left thyroid lobe without apparent signs of regional lymphadenopathy.

**Figure 3.**
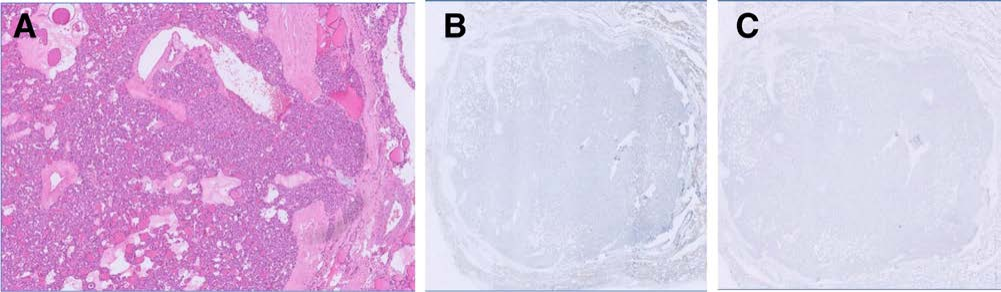
(A) Minimally invasive follicular thyroid carcinoma (FTC) with complete encapsulation, no vascular, lymphatic, or perineural invasion, no extrathyroid extension. (B) Negative immunohistochemistry for BRAF (clone: VE1, Ventana, Oro Valley, AZ, USA). (C) Negative Immunohistochemistry for NRAS Q61R (clone: RBT-NRAS, Bio SB, Goleta, CA, USA).

Further molecular genetic testing of FNA samples stored in CytoRich Red Collection Fluid (Thermo Fisher Scientific, Waltham, MA, USA) was conducted. The isolated DNA was quantified using Qubit™ dsDNA HS Assay Kit (Thermo Fisher Scientific, Waltham, MA, USA), and mutation-specific qPCR experiments were performed with ThyroSCAN Cancer Diagnostics Kit (Quak BioTechnology, Taiwan) for the detection of five single nucleotide variants (*BRAF* V600E, *NRAS* Q61R, *NRAS* Q61K, *HRAS* Q61R, *HRAS* Q61K). The *BRAF* V600E mutation was detected with a cycle threshold (Ct) value of 24.4, falling below the predetermined cutoff Ct value of 25 based on the standard curve method, resulting in the positive identification of *BRAF* V600E and raising concern for PTC. A preoperative neck computed tomography scan showed a 3.8-cm mass situated within the left thyroid lobe, while no regional lymphadenopathy was identified (Fig. 2). Therefore, the decision was made to proceed with a left lobectomy along with central lymph node dissection, and the patient underwent the procedure smoothly without any adverse events. The histological examination revealed a 3.7 × 3.5 × 1.5 cm tumor composed of solid pattern of microfollicles with complete encapsulation exhibiting minimal capsular invasion in one focus while showing no evidence of vascular or lymphatic invasion, nor perineural invasion, and lacking the nuclear characteristics typically associated with PTC, such as multifocal nuclear enlargement, nuclear overlapping, chromatin clearing, and nuclear grooves. Additionally, immunohistochemical analysis demonstrated negative staining for BRAF (clone: VE1, Ventana, Oro Valley, AZ, USA) and NRAS Q61R (clone: RBT-NRAS, Bio SB, Goleta, CA, USA) (Fig. 3). The final pathology report indicated minimally invasive FTC, staged as pT2N0M0.

**Figure 4.**
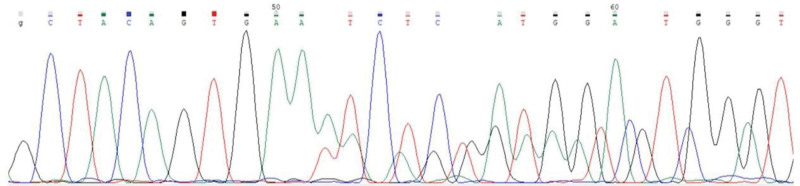
Electropherogram of the duplicated sequence following GTGAA. One represents the mutated allele with a nucleotide change: c.1794_1802dup (p. V600_K601insNTV), while the other corresponds to the wild-type allele.

For the discrepancy of preoperative qPCR and immunohistochemical staining, formalin-fixed paraffin-embedded tissue samples were subjected to perform Sanger sequencing of *BRAF* exon 15, uncovering a nucleotide duplication within the coding sequence spanning from 1794 to 1802, resulting in the protein alteration identified as V600_K601insNTV (Fig. 4). Although the final pathology results differed from the initial impression, the patient still appreciated that the malignancy was treated based on preoperative molecular genetic testing.

## 3. Discussion

We present a case featuring an indeterminate thyroid nodule whose FNA molecular testing showed *BRAF* V600E positivity, whereas, the final pathology surprisingly revealed a follicular carcinoma. Further molecular analysis of pathologic samples unveiled a rare *BRAF* V600_K601insNTV mutation, previously reported in only one case of follicular variant of PTC.^[10]^ To our best knowledge, this is the first reported case of FTC with *BRAF* V600_K601insNTV mutation. The prevalence of thyroid cancers with rare *BRAF* mutations is summarized in Table 2.

**Table 2:** Summary of thyroid carcinomas with rare *BRAF* mutation.

Nucleotide change	Protein change	Diagnosis	Histologic variant	Reported cases	Reference
c.1801A > G	p.K601E	PTC FTC FA Adenoma/goiter PDTC	MicroPTC FV-PTC TSV-PTC CV-PTC N/A N/A N/A N/A N/A	5 57 2 2 69 11 1 4 1	[10–22]
c.1799_1801delTGA	p.V600_K601delinsE	PTC	TSV-PTC CV-PTC FV-PTC HNV-PTC N/A	5 4 5 1 1	[10,14,16,18,23,24]
c.1798_1811del; c.1798_1799ins	p.T599I-V600_R603del	PTC	FV-PTC	2	[14,25]
c.1794_1795insGTT	p.A598_T599insV	PTC N/A	CV-PTC FV-PTC N/A N/A	1 1 1 1	[10,18,26–28]
c.?	p.VKSR600-3delT599I	PTC	FV-PTC	2	[13]
c.?	p.T599-V600delTinsIYI	PTC	FV-PTC	1	
c.1775_1795dup	p.I592_A598dup	PTC	FV-PTC	1	[10]
c.1745_1795dup	p.I582_A598dup	PTC	FV-PTC	1	
c.1781_1798dup	p.D594_T599dup	PTC	FV-PTC	1	
c.1798_1801delGTGA	p.V600_K601delinsQ	PTC	FV-PTC	1	
c.1803_1814de	p.K601_S605delinsN	PTC	FV-PTC	1	
c.1794_1802dup	p.V600_K601insNTV	PTC	FV-PTC	1	
c.1795_1797dupACA	p. T599dup	PTC	CV-PTC FV-PTC	1 1	[16]
c.1799_1814 > A	V600_S605 > D	PTC	CV-PTC	1	
c.1798_1810delinsA	p.V600_W604 > R	PTC	CV-PTC	1	
c.?	p.S614T	PTC	FV-PTC	1	[17]
c.?	p.K601_S602 > T	PTC	FV-PTC	1	
c.?	p.V600_W604 > DL	PTC	FV-PTC	1	
c.?	p.T599E	PTC	FV-PTC	1	
c.?	p.ΔV600	PTC	FV-PTC	1	
c.[1797A > G; 1799T > A]	p.[(=; V600R)]	PTC	CV-PTC	3	[18]
c.1797_1798insGAGACTACA	p.T599_V600insETT	PTC	FV-PTC	1	
c.[1799T > A; 1801_1812del]	p.[(V600E; K601_W604del)]	PTC	CV-PTC	1	
c.[1770_1795dup26; 1795_1796insA]	p.[K591_A598dup; A598_T599insK]	PTC	CV-PTC	1	
c.[1796C > G; 1799 T > A]	p.[(T599R; V600E)]	PTC	CV-PTC	1	
c.1794_1796del	p.T599del	FTC	N/A	1	[29]
c.1794_1796insTAC	p.T599dup	PTC	N/A	1	
c.1790T > A	p.L597Q	PTC	N/A	1	[30]
c.1796C > T	p.T599I	PTC	FV-PTC	1	[20]
c.1798inTAC	p.T599dup	PTC	FV-PTC	1	
c.C1796T; 1798_1799insCTT	p.T599I; V600delinsAL	PTC	TSV-PTC	1	[31]
c.1798delinsTACA	p.V600delinsYM	PTC	N/A	4	[32]
c.1793C > T	p. A598V	PTC	FV-PTC	1	[33]
c.1799_1814delinsATGT	p.V600_S605delinsDV	PTC	FV-PTC	1	[34]
c.?	p. G474R	PTC	FV-PTC	1	[21]
c.1834C > T	p.Q612	PTC	N/A	2	[22]
c.1778G > A	p.G593D	PTC	N/A	1	
c.1795_1796insTAA	p.A598_T599insI	PTC	N/A	1	
c.1795_1796ins27	p.A598_T599insKKIGDFGLA	PTC	N/A	1	
c.1796_1797insTAC	p.T599_V600insT	N/A	N/A	1	
c.1797_1798ins9	p.T599_V600insETT	PTC	N/A	1	
c.1798_1799ins18	p.T599_V600insDFGLAT	PTC	N/A	1	
c.?	p.K601del	PTC	N/A	3	
c.1801_1803delAAA	p.K601del	PTC	N/A	1	
c.?	p.V600_W604del	PTC	N/A	3	
c.1802_1813del	p.K601_W604del	PTC	N/A	1	
c.1796_1809delinsTC	p.T599_R603delinsI	PTC	N/A	4	
c.1796_1798delinsTAGCTT	p.T599_V600delinsIAL	PTC	N/A	2	
c.?	p.T599_V600 > IYI	PTC	N/A	1	
c.1794_1802dup	p. V600_K601insNTV	FTC	N/A	1	Our case

CV-PTC = classic variant of papillary thyroid carcinoma, FA = follicular adenoma, FTC = follicular thyroid carcinoma, FV-PTC = follicular variant of papillary thyroid carcinoma, HNV-PTC = hobnail variant of papillary thyroid cancer, PDTC = poorly differentiated thyroid carcinoma, PTC = papillary thyroid carcinoma, TSV-PTC = trabecular-solid pattern of growth papillary thyroid carcinoma.

Previous studies have identified numerous rare *BRAF* mutations, with *BRAF* K601E being the most prevalent, particularly associated with follicular variant of PTC, while the V600_K601delinsE mutation ranks as the second most common variant.^[10–13]^ Additionally, another infrequent *BRAF* mutation, *BRAF* G469A, is barely observed in thyroid tumors but primarily occurs in malignant struma ovarii with thyroid-type carcinoma morphology.^[35]^ Based on current literature, thyroid carcinomas harboring rare *BRAF* mutations are generally linked to less aggressive tumor behavior, characterized by unifocality, complete encapsulation, intrathyroidal presentation with a low incidence of lymph node metastases, and less advanced tumor staging at diagnosis, consistent with the findings in our patient.^[10]^

The pathogenesis of PTC is influenced by the mitogen-activated protein kinase (MAPK) pathway, which plays a crucial role in regulating cellular proliferation, differentiation, and programmed cell death.^[36]^ Within this pathway, the predominant driver mutation observed in PTC occurs in the 15th exon of the *BRAF* gene, resulting in a substitution of valine with glutamate at codon 600, known as *BRAF* V600E.^[26]^ Moreover, *RAS* mutations, mutually exclusive with *BRAF* V600E, also trigger the activation of the MAPK pathway.^[37]^ Despite both involvement in the MAPK pathway, *BRAF* and *RAS* mutations lead to different degrees of activation within the signaling cascade.^[36,38]^ pERK1/2 serves as an indicator of MAPK pathway activity, with higher expression levels observed in *BRAF*-mutated tumors compared to *RAS*-mutated tumors.^[36,38]^ This disparity may elucidate the association of *BRAF* V600E with a more unfavorable prognosis, including heightened disease recurrence, increased tumor aggressiveness, extrathyroidal spread, and the development of local and distant lymph node metastases.^[39]^ Conversely, *RAS* mutations are often associated with less aggressive behavior, frequently presenting as tumors with a follicular growth pattern, encapsulation, minimal invasiveness, and a reduced likelihood of recurrence.^[38,40]^ Given that all *BRAF* mutations other than *BRAF* V600E exhibit *RAS*-like behavior, it may explain why thyroid cancers with rare *BRAF* mutations exhibit an indolent nature and a higher frequency of the follicular variant.^[36]^ While our case report unveiled a novel *BRAF* mutation in FTC displaying *RAS*-like characteristics, additional research is required to clarify the involvement of other pathways implicated in the pathogenesis of PTC with rare *BRAF* mutations, that is, the phosphatidylinositol-3 kinase/serine-threonine protein kinase AKT signaling pathway and PAX8/peroxisome proliferator-activated receptor γ rearrangement. Furthermore, the effects of V600_K601insNTV on the MAPK signaling pathway and its responsiveness to *BRAF* inhibitors remain unexplored and warrant further investigation.

Molecular markers have emerged as a promising approach in cases of indeterminate thyroid FNA specimens, offering crucial insights to guide decisions regarding primary surgical treatment.^[5,41]^ The prevalence of *BRAF* V600E mutation differs in different subtype of thyroid cancer, depending on the detection method and size of study cohort. The *BRAF* V600E mutation is detected in approximately 30-70% of PTC cases and 30% to 40% of anaplastic thyroid carcinoma cases.^[39,40,42]^ Recent reports showed *BRAF* V600E mutation was detected in around 10% of FTC according to the 2017 WHO classification, but not detected in FTC according to the 2023 WHO classification, in which the oncocytic carcinomas of the thyroid are distinguished from FTC.^[43,44]^ Given its marked specificity, thyroidectomy is recommended when *BRAF* V600E mutation is present.^[45,46]^ The recognition of *BRAF* mutation as a potential predictive indicator for occult metastasis also prompts consideration of elective neck dissection in region VI.^[45,46]^ Nonetheless, implementing neck dissection based on preoperative identification of the *BRAF* V600E mutation in cN0 PTC still remains controversial, primarily due to concerns regarding potential surgical complications and the limited positive predictive value of disease recurrence.^[47]^ In retrospect, the necessity of the central lymph node dissection procedure for our patient may warrant reconsideration. However, it is essential to highlight that despite the potential for false positives, preoperative FNA cytology molecular genetic analysis significantly contributes to the early intervention of malignant thyroid nodules.

In this study, the Ct value of *BRAF* V600E detected by mutation-specific qPCR was 24.4, which is close to the cutoff Ct value of 25. Although the false positive rate of qPCR detection methods ranges from 0.08% to 5.4%,^[48]^ our results certainly need further validation. *BRAF* V600E mutation detection methods have been greatly developed, including digital PCR. Locked nucleic acid probe-based digital PCR can be used to further validate our results, as it has been shown to accurately detect *BRAF* V600E variants with higher sensitivity than immunohistochemistry and Sanger sequencing.^[49]^ In addition, qPCR and digital PCR methods are mainly used to detect known biomarkers and are limited in discovering new mutations.

## 4. Conclusions

The case presented highlights the critical need to thoroughly evaluate molecular genetic testing results in thyroid nodule FNA cytology. While the *BRAF* V600E mutation is commonly linked to PTC, its presence does not exclude other types of thyroid malignancies. Additionally, the identification of a rare *BRAF* mutation in our case, previously unreported in FTC, exhibited an indolent behavior consistent with its *RAS*-like characteristics.

## Acknowledgments

All authors have participated in clinical care of the patient and worked on the literature review and modification of our manuscript. We appreciated all the efforts contributed by all the authors of this article.

## Author contributions

**Writing—original draft:** Po-Sheng Lee

**Conceptualization:** Jui-Yu Chen, Li-Hsin Pan, Chin-Sung Kuo

**Methodology:** Jui-Yu Chen, Jen-Fan Hang

**Project administration:** Chii-Min Hwu

**Resources:** Chii-Min Hwu, Jen-Fan Hang

**Supervision:** Chii-Min Hwu, Chin-Sung Kuo

**Validation:** Chii-Min Hwu

**Investigation:** Jen-Fan Hang

**Writing—review & editing:** Chin-Sung Kuo
